# The impact of alternative pricing methods for drugs in California Workers’ Compensation System: Fee-schedule pricing

**DOI:** 10.1371/journal.pone.0197449

**Published:** 2018-05-25

**Authors:** Leslie Wilson, Fatema A. Turkistani, Wei Huang, Dang M. Tran, Tracy Kuo Lin

**Affiliations:** 1 Department of Clinical Pharmacy, University of California San Francisco, San Francisco, California, United States of America; 2 Department of Clinical and Hospital Pharmacy, College of Pharmacy, Taibah University, Al-Madinah, Saudi Arabia; 3 Department of Social Policy, London School of Economics and Political Science, London, United Kingdom; National Yang-Ming University, TAIWAN

## Abstract

**Introduction:**

California’s Workers’ Compensation System (CAWCS) Department of Industrial Relations questioned the adequacy of the current Medi-Cal fee-schedule pricing and requested analysis of alternatives that maximize price availability and maintain budget neutrality.

**Objectives:**

To compare CAWCS pharmacy-dispensed (PD) drug prices under alternative fee schedules, and identify combinations of alternative benchmarks that have prices available for the largest percentage of PD drugs and that best reach budget neutrality.

**Methods:**

Claims transaction-level data (2011–2013) from CAWCS were used to estimate total annual PD pharmaceutical payments. Medi-Cal pricing data was from the Workman’s Compensation Insurance System (WCIS). Average Wholesale Prices (AWP), Wholesale Acquisition Costs (WAC), Direct Prices (DP), Federal Upper Limit (FUL) prices, and National Average Drug Acquisition Costs (NADAC) were from Medi-Span. We matched National Drug Codes (NDCs), pricing dates, and drug quantity for comparisons. We report pharmacy-dispensed (PD) claims frequency, reimbursement matching rate, and paid costs by CAWCS as the reference price against all alternative price benchmarks.

**Results:**

Of 12,529,977 CAWCS claims for pharmaceutical products 11.6% (1,462,814) were for PD drugs. Prescription drug cost for CAWCS was over $152M; $63.9M, $47.9M, and $40.6M in 2011–2013. Ninety seven percent of these CAWCS PD claims had a Medi-Cal price. Alternative mechanisms provided a price for fewer claims; NADAC 94.23%, AWP 90.94%, FUL 73.11%, WAC 66.98%, and DP 14.33%. Among CAWCS drugs with no Medi-Cal price in PD claims, AWP, WAC, NADAC, DP, and FUL provided prices for 96.7%, 63.14%, 24.82%, 20.83%, and 15.08% of claims. Overall CAWCS paid 100.52% of Medi-Cal, 60% of AWP, 97% of WAC, 309.53% of FUL, 103.83% of DP, and 136.27% of NADAC.

**Conclusions:**

CAWCS current Medi-Cal fee-schedule price list for PD drugs is more complete than all alternative fee-schedules. However, all reimbursement approaches would require combinations of pricing benchmarks. We suggest keeping primary reimbursement at 100% of Medi-Cal and for drugs without a primary Medi-Cal price calculating the maximum fee as 60% of AWP and then 97% of WAC. Alternatively, we suggest using NADAC as a primary fee-schedule followed by either 60% AWP and 97% WAC or AWP-40% for drugs with no NADAC price. Fee-schedules may not offer the best price and a formulary approach may provide more flexibility.

## Introduction

The net spending on prescription drugs in the United States has been rising more rapidly than the Consumer Price Index for all items,[1] increasing by 20% between 2013–2015[2]. High drug costs affect all sectors of the healthcare system including the Workers’ Compensation System (WC).[3] WC is a state-based liability insurance program that most employers are required to pay into in order to fund the medical bills, recovery costs and partial missed wages when an employee is injured at work. The WC insurance and medical care are separate from a companies’ health insurance and are usually provided free-of-charge to an injured worker, unlike services provided under the employee’s standard employment health insurance. In 2014, pharmacy costs accounted for 18% of total WC insured medical spending; paying an average $1,583 on prescription drugs for every injured worker.[4] In the United States there is a general lack of transparency in drug prices. It is not clear that the fee-schedule approach, such as that used by many worker’s compensation systems, gives payers the best prices^5^. For example, the National Council on Compensation Insurance, Inc. (NCCI) found that WC insurers paid an average of 125% of AWP compared with 72% paid by private negotiated insurance contracts.[5] In general, payers arrive at the drug prices they pay in a number of ways; including contract pricing with or without the use of a fee-schedule of allowed drugs, or based on government regulation of prices for Medicare, Medicaid, CAWCS, and Veterans administration programs. All of these methods of drug pricing are then based on a specific price per drug using one of the available lists of calculated prices discussed in detail later in this paper.

California is the largest workers’ compensation system in the U.S, comprising approximately 25% of the entire nations’ workers’ compensation premiums.[6] Beginning in 2004, the state required the CAWCS Official Medical Fee-Schedule (OMFS) reimburse both brand and generic drugs at 100% of Medi-Cal (California’s Medicaid) outpatient pharmacy fee-schedule, as its primary payment system. This practice resulted in tying CAWCS’s reimbursement to the state- and federal-based rebate negotiations made external to the CAWCS itself.[7] Therefore their changes were not transparent to CAWCS pricing needs. For drugs not covered by a Medi-Cal payment system (such as for repackaged drugs), the secondary payment system was the maximum fee paid, calculated as 83% of AWP of the lowest therapeutically equivalent drug (calculated on per unit basis) + $7.25 to pharmacists as a dispensing fee ($8.00 if the patient is in a skilled nursing or intermediate care facility).[8] After adapting to the new Medi-Cal-based reimbursement system, drug spending by CAWCS declined by 27% from 2004 to 2007, but then increased by 42% from 2007 to 2013.[9] Medi-Cal initially was a good fit as a pricing benchmark for CAWCS because outpatient prescription drug coverage included all federally required drug classes, also required by CAWCS regulations, yet had strong price controls. Medi-Call controlled their own prescription unit price drug spending primarily by maximizing federal and state supplements rebates.[8] But as more Medi-Cal beneficiaries moved to managed care plans, Medi-Cal also implemented utilization controls and co-payments, not allowed under the CAWCS fee-schedule system regulations. The drugs used by managed care beneficiaries were also not included on the Medi-Cal primary fee-schedule price list used to price the CAWCS fee-schedule drugs. In addition, the Medicare Part D benefit created by the Medicare Modernization Act moved primary coverage for outpatient prescription drug coverage from Medi-Cal to Medicare for all dual-eligible beneficiaries. Despite Medi-Cal continuing to pay for drugs in categories excluded from Part D and available to other Medi-Cal beneficiaries, CAWCS began seeing different and fewer drugs on the fee-for-service based Medi-Cal list used as the primary payment system for its drugs. This required CAWCS to increasingly pay based on the higher cost AWP-based secondary payment system.

Other changes also began to erode the stability of Medi-Cal as a pricing system for CAWCS. For example, under ACA implementation in 2010, Medicaid payments were required to be at an aggregate upper limit based on actual acquisition cost (AAC) as all payers of drugs wanted to move away from an AWP-based pricing toward this market-based price approach. But as California began the process of transitioning to an AAC benchmark, it was put on hold by California’s Department of Health Care Services (DHCS)[10] likely, in part, because CMS began collecting and publishing a National Average Drug Acquisition Cost (NADAC) to provide a national reference file to assist state Medicaid programs in evaluating their reimbursement. Currently Medi-Cal (and therefore CAWCS) has not moved from the AWP-based pricing and still defines the pharmacy rate paid as the lower of (1) AWP– 17%; (2) FUL; or (3) the maximum allowable ingredient cost (MAIC).[11] But all of these changes have resulted in a lack of California Workers’ Compensation fee-schedule prices for an increasing number of NDCs as well as delays in updating Medi-Cal price data.[12]

CAWCS requested this analysis to address their doubts about the adequacy of the current Medi-Cal pricing as its primary fee-schedule benchmark. They knew that as the number of drug claims without a Medi-Cal price increased and required the higher secondary AWP-based prices, they were no longer getting the lowest prices available for drugs. The goal of this study was to determine alternatives for pricing their current fee-schedule that maximize price availability and maintain access and budget neutrality. Alternative pricing benchmarks used by both government (Medi-Cal, FUL and NADAC) and also by the private sector (AWP, WAC, and DP) were assessed. Since there is little transparency in the drug prices across each benchmark, the Medi-Cal-based prices over three years of CAWCS drug claims were used to demonstrate how Medi-Cal payments compared with other government and private pricing benchmarks that are the hallmarks of fee-schedule pricing methods.

This analysis compared CAWCS utilization and prices across years and used CAWCS claims to compare retail pharmacy drug prices under alternative fee-schedules. Efficient alternative benchmarks that had the largest number of listed prices were also identified. In addition, the prices of the same set of CAWCS drugs across both government and private pricing methods was compared to elucidate their price transparency nationally for others using fee-schedule drug pricing.

## Materials and methods

The study protocol was exempted by the institutional review board (IRB) of University of California, San Francisco. Claims data with no identifiers were used so no informed consent was required from patients.

### Data sources

Several datasets were used to analyze drug prices within a pharmaceutical fee-schedule approach to pricing: 1) the Workers’ Compensation Information System (WCIS), 2) A Medi-Span data set including drug prices for five different private pricing mechanisms, and 3) Medi-Cal and NADAC drug prices available by CMS on-line. Claims level data from WCIS covering pharmacy transaction costs were used to estimate total annual pharmacy-dispensed (PD) drug utilization and costs from January 1, 2011 through December 31, 2013. WCIS data was divided into four claims data subsets; physician-dispensed and administered drugs, compounded drugs, medical foods, and physician-dispensed (PD) drugs. Only the PD drug claims data was used in this study because any regulatory changes arising from our analysis are unique to PD drugs. Claims records included the date of service, billed amount, paid amount, claim identifiers, patient identifiers, NDC, dispensing agent, drug dispensed, dispensing fee, quantity dispensed, and other supporting information. Service adjustment codes indicated the reason for any discrepancy between billed and paid amounts, and allowed us to remove duplicate and zero billed records appropriately. Each drug claim had an 11-digit NDC which identified the manufacturer, drug dispensed, strength of the drug, and the wholesale package size. Drug therapeutic class was assigned to each line item by pharmacist experts in the field. Weekly Medi-Cal datasets of drug prices were obtained from WCIS to verify the drug prices in the claims data set. CAWCS annual PD claims were then repriced with each different pricing benchmark as described in Table 1. To provide greater transparency across these different pricing benchmarks, each pricing alternative was compared with the sample portion of matched claims that had an available list price in both CAWCS and that particular pricing benchmark. Two different types of benchmarks are used; government pricing fee-schedules (FUL and NADAC) whose prices are available on a government website, and private pricing benchmarks (AWP, WAC and DP) whose fee-schedule prices must be purchased (Table 1). The Medi-Span dataset included prices by NDC for five different pricing mechanisms used in this study: AWP, WAC, DP, FUL, and NADAC (Table 1).[13] CAWCS drugs were repriced relative to other fee- schedules for each NDC, using the unit of quantity dispensed and the date of service. All FUL, and NADAC prices were converted to 2011, 2012, and 2013 US dollars using the US Consumer Price Index (CPI-U) average for prescription drugs.[14] Our analysis excluded any claims with a missing quantity dispensed, a pending payment status, a claim duplication, and negative or zero total paid amounts.

**Table 1:** Description and availability of alternative fee-schedule pricing benchmarks.

TYPE OF PRICING BENCHMARK	DESCRIPTION	AVAILABILITY *
**Government Benchmarks**		
Federal Upper Limit (FUL)	CMS calculates FUL as 175% of weighted average of the most recently reported monthly average manufacturer price (AMP) for multiple source drug products that are available in retail community pharmacies.[28]	Manufacturers report monthly AMP to CMS and they calculate FUL. Medi-Cal FUL based prices are reported on their website at Data.Medicaid.gov [https://data.medicaid.gov/Drug-Pricing-and-Payment/ACA-Federal-Upper-Limits/yns6-zx8k]
National Average Drug Acquisition Cost (NADAC)	Provide the average drug market prices paid in US as provided by periodic CMS conducted surveys of randomly selected, retail community pharmacy prices nationwide.[17]	NADAC prices are available on the Medicaid.gov website (at Data.Medicaid.gov) from November, 2013 to the present. [https://www.medicaid.gov/medicaid/prescription-drugs/pharmacy-pricing/index.html] and
**Private Benchmarks**		
Average Wholesale Price (AWP)	A self-reported manufacturer price, but AWP does not really represent the actual wholesale price paid by pharmacies after discount and rebates.[29]	Only available for purchase*
Wholesale Acquisition Cost (WAC)	The list price paid by the wholesaler or a direct purchaser for drugs purchased from a drug manufacturer before any discounts, rebates, purchasing allowances or other forms of economic consideration.[30]	Only available for purchase*
Direct Price (DP)	The list price used for invoices between drug manufacturers and pharmacies or providers. Price before any discounts, rebates, purchasing allowances or other forms of economic consideration.[31]	Only available for purchase*

*Note: Private Benchmark prices are only available for purchase from: Medi-Span from Wolters Kluwer, [http://www.wolterskluwercdi.com/drug-data/], Redbook from Truven Health Analytics and Online from Micromedix, http://www.micromedixsolutions.com/, and First Databank [http://www.fdbhealth.com/fdb-medknowledge-drug-pricing/]. They provide these prices for only those drugs that they can obtain from others so do not include prices for all NDCs. Medi-Span provided our drug prices for the privately available benchmarks

### Sample

CAWCS reimburses drugs dispensed by both pharmacies and by physicians. Despite being reimbursed by the same fee-schedule, they each have a unique set of policies and regulations governing their reimbursement and pricing. This study sample included only CAWCS prescription drugs dispensed at the pharmacy retail (PD) level.

### Data analysis

To compare the alternative pricing methods the reimbursed PD prescriptions were evaluated at the transaction level, and reported as PD claims frequency, reimbursement rate, and total & average paid costs across years. Estimated costs for different pricing benchmarks were compared to the actual amount paid by CAWCS. We matched each claim line in CAWCS by NDC and the dates of service to the corresponding unit price of an alternative price benchmark. Each unit price from different price benchmarks was multiplied by the quantity unit dispensed in CAWCS. For PD claims that had no prices under different primary price benchmarks, we calculated the percentage and total amount paid by CAWCS for drugs with no listed primary benchmark price. The results were compared across different primary and secondary price benchmarks used in our study. Comparisons of CAWCS paid amounts with each alternative price benchmark included a paired sample of the same drugs by NDC and amount. Descriptive statistics and Analysis of variance (ANOVA) were performed to assess statistical differences in the change in drug expenditure over time. The statistical significance level was set at 0.05. All statistical analyses were conducted using Stata MP Version 14 and Microsoft Excel 2013.[15]

## Results

Between 2011 and 2013, there were 20,373,477 total claims submitted (billed) to CAWCS. Of these, 12,529,977 claims were for pharmaceutical products. Claims billed for pharmaceutical products dispensed by pharmacies (PD) accounted for 11.6% (1,462,814) of all pharmaceutical product claims. Numbers of PD claims billed of total billed claims decreased over the years; 595,849 (40.73%) claims in 2011, 476,203 (32.6%) claims in 2012, and 390,762 (26.7%) claims in 2013. Of these billed claims, not all were reimbursed due to various types of errors in billing or decisions not to pay the claim. Reimbursed (paid) PD claims in CAWCS represented approximately 69% (1,011,628) out of 1,462,814 total PDs billed claims in CAWCS across all 3 years (Table 2). Only reimbursed (paid) claims were included in our repricing analysis as this represents amounts paid by CAWCS.

**Table 2:** Percent of CAWCS claims and differences in paid amount for the percent of CAWCS retail pharmaceuticals with NDC prices under current workers compensation Medi-Cal fee-schedule and each alternative pricing benchmark by year.

Year		% CAWCS claims with drug prices under different pricing benchmark	Amount paid by CAWCS for claims with drug prices under each pricing benchmark	Total Estimated Costs (EC) using different pricing benchmarks for CAWCS claims with drug prices available	The difference between amount paid by CAWCS and Estimated Costs by different pricing benchmark	% Change (amount paid by CAWCS/ EC by different pricing benchmark)	Formula to equalize the amount paid by CAWCS (WC) and EC when pricing by each pricing benchmark for Matched NDC's to maintain budget neutrality *
**2011**	*MC*	98.21%	$55,022,480	$56,213,969	-$1,191,489	97.88%	**WC** = MC—2.12%
**2012**		96.48%	$31,765,886	$32,661,798	-$895,912	97.26%	**WC** = MC—2.74%
**2013**		96.30%	$22,869,387	$22,751,290	$118,097	100.52%	**WC** = MC + 0.52%
**Overall**		97.29%	$109,657,753	$111,627,057	-$1,969,304	98.23%	**WC = MC– 1.77%**
**2011**	*AWP*	86.60%	$44,419,718	$65,456,801	-$21,037,083	67.86%	**WC** = AWP—32.14%
**2012**		94.27%	$33,028,284	$53,547,001	-$20,518,717	61.68%	**WC** = AWP—38.32%
**2013**		96.41%	$23,988,919	$39,730,649	-$15,741,730	60.38%	**WC** = AWP—39.62%
**Overall**		90.94%	$101,436,921	$158,734,451	($57,297,530)	63.90%	**WC = AWP– 36.10%**
**2011**	*WAC*	58.46%	$36,889,531	$34,953,066	$1,936,465	105.54%	**WC** = WAC + 5.54%
**2012**		71.84%	$29,185,546	$31,136,239	-$1,950,693	93.73%	**WC** = WAC—6.27%
**2013**		80.12%	$21,961,625	$22,608,304	-$646,679	97.14%	**WC** = WAC– 2.86%
**Overall**		66.98%	$88,036,702	$88,697,609	-$660,907	99.25%	**WC = WAC– 0.745%**
**2011**	*FUL*	71.89%	$31,096,368	$10,885,684	$23,542,460	285.66%	**WC** = FUL + 216.27%
**2012**		73.44%	$19,107,542	$6,458,913	$12,648,629	295.83%	**WC** = FUL + 195.83%
**2013**		75.56%	$13,631,376	$4,403,915	$9,227,461	309.53%	**WC** = FUL + 209.53%
**Overall**		73.11%	$63,835,286	$21,748,512	$42,086,774	293.52%	**WC = FUL + 193.52%**
**2011**	*DP*	15.22%	$10,924,509	$10,009,569	$914,940	109.14%	**WC** = DP + 9.14%
**2012**		13.85%	$7,281,057	$6,881,417	$399,640	105.81%	**WC** = DP + 5.81%
**2013**		12.95%	$5,656,545	$5,447,692	$208,853	103.83%	**WC** = DP + 3.83%
**Overall**		14.33%	$23,862,111	$22,338,678	$1,523,433	106.82%	**WC = DP + 6.82%**
**2011**	*NADAC*	94.76%	$50,774,891	$40,104,114	$10,670,777	126.61%	**WC** = NADAC + 26.61%
**2012**		93.31%	$29,008,123	$21,128,000	$7,880,123	137.30%	**WC** = NADAC + 37.30%
**2013**		94.36%	$21,309,744	$15,637,534	$5,672,210	136.27%	**WC** = NADAC + 36.27%
**Overall**		94.23%	$101,092,758	$76,869,648	$24,223,110	131.51%	**WC = NADAC +31.51%**

**CAWCS (WC),** California Workers’ Compensation System; **MC**, Medi-Cal; **AWP**, Average Wholesale Price; **WAC**, Wholesale Acquisition Cost; **DP**, Direct Price; **FUL,** Federal Upper Limit; **NADAC,** National Average Drug Acquisition Cost; **EC**, Estimated Costs; **NDC’s**, National Drug Code

This table assessed

• The percent of drugs with NDC prices under each pricing benchmarks and the paid cost of these drugs compared to current CAWCS Medi-Cal paid claims.

• The formula * to maintain budget neutrality if switching drug prices from current CAWCS Workman’s’ Compensation (WC) fee-schedule to each Pricing Benchmark

Fee-schedule for a sample of matched NDCs

### Current Medi-Cal based fee-schedule drug use and costs

Over the three years, the number of PD drug claims paid (CP) decreased from 495,759)) in 2011, 308,647 in 2012, and 207,222 CP in 2013 (P = 0.08) (Table 3). Between 2011 and 2013, the cost (or actual amount paid) for PD prescription drugs in workers’ compensation for all California employers was over $152 million; decreasing annually from $63.9 million in 2011, to $47.9 million in 2012, and $40.6 million in 2013 (Table 2). The mean cost paid per claim, however, increased over the time; $128.9 in 2011, $155.3 in 2012, and $197.2 in 2013 (data not shown).

**Table 3:** Percent of CAWCS claims and differences in paid amount for the percent of CAWCS retail pharmaceuticals with no NDC prices under current workers compensation Medi-Cal fee-schedule and each alternative pricing benchmark by year.

% CAWCS claims with drug prices under different pricing benchmark			Amount paid by CAWCS for claims with drug prices under each pricing benchmark	Total EC by different pricing benchmark for CAWCS claims with drug prices available	The difference between amount paid by CAWCS and EC by different pricing benchmark	% Change (amount paid by CAWCS/ EC by different pricing benchmark)	% CAWCS claims without drug Prices under different pricing benchmark	Amount paid by CAWCS for claims without drug prices under each pricing benchmark	% (Amount paid by CAWCS for claims with no drug prices under each pricing benchmark / Amount paid by CAWCS for all claims)	The correlation between the amount paid by CAWCS over estimated costs by different pricing benchmark for Matched NDC's *
**2011**	*AWP*	96.91%	$1,156,375	$1,771,929	-$615,554	65.26%	3.09%	$16,303	1.39%	WC = AWP—34.74%
**2012**		96.10%	$2,540,983	$8,484,421	-$5,943,438	29.95%	3.90%	$66,837	2.56%	WC = AWP—70.05%
**2013**		97.31%	$1,628,278	$7,429,703	-$5,801,425	21.92%	2.69%	$68,846	4.06%	WC = AWP—78.08%
**Overall**		96.70%	$5,325,626	$17,686,054	-$12,360,428	30.11%	3.30%	$151,983	2.77%	WC = AWP—69.89%
**2011**	*WAC*	43.04%	$506,165	$529,984	-$23,819	95.51%	56.96%	$666,511	56.84%	WC = WAC—4.49%
**2012**		69.91%	$1,916,630	$5,891,280	-$3,974,650	32.53%	30.09%	$691,187	26.50%	WC = WAC—67.47%
**2013**		76.83%	$1,375,242	$4,990,529	-$3,615,287	27.56%	23.17%	$321,875	18.97%	WC = WAC—72.44%
**Overall**		63.14%	$3,798,036	$11,411,794	-$7,613,758	33.28%	36.86%	$1,679,576	30.66%	WC = WAC -66.72%
**2011**	*FUL*	32.79%	$263,418	$99,874	$163,544	263.75%	67.21%	$909,256	77.54%	WC = FUL + 163.75%
**2012**		9.05%	$82,422	$33,872	$48,550	243.33%	90.95%	$2,525,396	96.84%	WC = FUL + 143.33%
**2013**		3.11%	$9,402	$3,906	$5,496	240.71%	96.89%	$1,687,718	99.45%	WC = FUL + 140.71%
**Overall**		15.08%	$355,242	$160,874	$194,368	220.82%	84.92%	$5,122,369	93.51%	WC = FUL + 120.82%
**2011**	*DP*	29.06%	$427,026	$441,140	-$14,114	96.80%	70.94%	$745,649	63.59%	WC = DP—3.20%
**2012**		14.48%	$532,365	$525,676	$6,689	101.27%	85.52%	$2,075,453	79.59%	WC = DP—1.27%
**2013**		20.28%	$364,977	$402,383	-$37,406	90.70%	79.72%	$1,332,147	78.49%	WC = DP—9.30%
**Overall**		20.83%	$1,324,362	$1,369,198	-$44,836	96.73%	79.17%	$4,153,247	75.82%	WC = DP—3.27%
**2011**	*NADAC*	35.59%	$449,534	$496,684	-$47,150	90.51%	64.41%	$723,141	61.67%	WC = NADAC—9.49%
**2012**		17.86%	$208,957	$230,548	-$21,591	90.63%	82.14%	$2,398,861	91.99%	WC = NADAC—9.37%
**2013**		22.19%	$237,787	$256,079	-$18,292	92.86%	77.81%	$1,459,335	85.99%	WC = NADAC—7.14% (2011kjf
**(2012–2013)**		14.82%	$896,278	$983,310	-$87,032	91.15%	75.18%	$4,581.332	83.64%	WC = NADAC -8.85%

**CAWCS (WC),** California Workers’ Compensation System; **MC**, Medi-Cal; **AWP**, Average Wholesale Price; **WAC**, Wholesale Acquisition Cost; **DP**, Direct Price; **FUL,** Federal Upper Limit; **NADAC,** National Average Drug Acquisition Cost; **EC**, Estimated Costs; **NDC’s**, National Drug Code

This table assessed

• The drug availability under each pricing benchmarks and estimate the cost of these drugs compared to CAWCS claims.

• The percentages of claims and amount paid for drugs that are not benchmarked under each pricing mechanism represent the calculated CAWCS based formulas using each pricing system to maintain budget neutral

• The percentages of claims and amount paid for drugs that are not benchmarked under each pricing mechanism represent the calculated CAWCS based formulas using each pricing system to maintain budget neutral

### Repricing primary benchmarks

The total and annual number, percent and costs of CAWCS claims were first estimated using each alternative fee- schedule (Table 2). On average, Medi-Cal, which CAWCS currently uses to price their drugs, had a price for 97% of the drugs CAWCS paid over the 3 years; (96.3% 199,561 claims out of 207,222) in 2013. The percentage of CAWCS fee-schedule NDCs paid in 2013 that had an AWP-based NDC price was 96.41% (199,778 claims out of 207,222), while 94.36% (195,526 claims out of 207,222) had a NADAC-based NDC price, 80.12% (166,016 claims out of 207,222), had a WAC-based price, 75.56% (156,572 claims out of 207,222) had an FUL-based price and only 13% (26,827 claims out of 207,222) had a DP in 2013 (Table 2). Next, the current amount paid by CAWCS annually was compared to the percent difference in and the total amount that would be paid for the same fee-schedule sample of NDCs when using each different pricing benchmark (Table 2). In 2013 CAWCS paid 60.38% of AWP estimated costs for the CAWCS drugs that also have a unit price under the AWP fee-schedule. For the CAWCS drugs that also have a unit price under each alternative fee-schedule, CAWCS paid 136.27% of NADAC estimated costs, 97.14% of WAC estimated costs, 309.53% of FUL estimated costs, and 103.83% of DP estimated costs (Table 2). The CAWCS based formulas varied by year for each pricing alternative that would be required to maintain budget neutrality (Table 2).

### Repricing secondary benchmarks

During the study period, an average of 3% (27,397 out of 1,011,628) of PD claims had no Medi-Cal prices. These prescriptions account for 4.76% (approximately $5 million out of $115 million) of PD prescription payments. The percentage of PD claims that had no Medi-Cal prices increased over the time, starting in 2011 when only 1.8% (8,876 claims out of 495,759) had no Medi-Cal based price. These claims accounted for over $1 million in prescription payments. In 2012 the pricing lack increased to 3.5% (10,860 claims out of 308,647) and $2.6 million in prescription payments, and in 2013 3.70% (7,661 claims out of 207,222) accounting for over $1.6 million in payments (S1 Dataset). A comparison of the percentage of claims with list prices available for each alternative pricing fee-schedule for only the fee-schedule sample of NDCs that had no CAWCS Medi-Cal list price showed that AWP had the largest percentage (96.7%) or (26,493 claims out of 27,397) and CAWCS paid 30% of AWP estimated costs for these drugs under the AWP fee-schedule (Table 3). WAC had the next most list prices available for those claims with CAWCS Medi-Cal list price (63.1%) or (17,298 claims out of 27,397) and CAWCS paid 33.3% of WAC for these drugs. The NADAC fee-schedule had list prices for only 24.8% (6,799 claims out of 27,397) of this sample; DP for only 20.8% (5,706 claims out of 27,397), and FUL for only 15% (4,131 claims out of 27,397) of this sample (Table 3).

The pricing implications of re-pricing the claims that have no Medi-Cal price along with the formulas required to reach budget neutrality for each alternative benchmark for this sample are presented in Table 3.

## Discussion

This study is unique as the first study that analyzed alternative price benchmarks for drugs dispensed by pharmacies using a fee-schedule pricing system such as used by the CAWCS.

Patterns of CAWCS drug use and costs over time: The analysis showed that the number of PD claims and total amount paid for PD claims by CAWCS consistently declined over the three years. Incomplete claims records might explain this decline. However, this is unlikely since we allowed at least 3 years for claims resolution. The decline in pharmacy-dispensed claims was more likely due to a shift of claims from the pharmacy to the physicians, who are allowed to and frequently dispense drugs within the workers’ compensation system. The National Council on Compensation Insurance, Inc. (NCCI) found that the WC physician-dispensed pharmaceuticals increased from 1.4 to 1.6 prescriptions between 2007 and 2011. Therefore, this increase likely continues and is offsetting the decrease in PD drug claims that our analysis shows.[16] Previous studies found that chain pharmacies are less willing than physicians to assume the risk of nonpayment, especially for first-time prescriptions. Early non-payment of treatments is common for patients injured at work. In the same study, pharmacies reimbursement uncertainties resulted in more workers being required to pay up-front and seek reimbursement later from workers compensation payers.[5] Therefore physician-dispensed pharmaceuticals can be helpful in this injured population with difficulty accessing retail pharmacy. Physician dispensing could act to enhance medication compliance[5] since reports show that as many as 20% of patients failed to get a prescription filled and 30% of patients did not obtain refills.[7] Physician-dispensed pharmaceuticals accounted for the majority of all pharmaceutical reimbursement in WC in our study and others,^5^ but will require different repricing strategies from the ones suggested here.

This added convenience of allowing physician dispensing likely comes at a price, however. In CAWCS, one study showed that the cost for PD medicines were three times more than similar medications dispensed by retail pharmacies.[5,17] Physicians may dispense repackaged medications that are not available in retail pharmacies, and these often have higher costs as well. Our analysis showed that this decline in total PD payments over time was accompanied by compensating increases over time in the PD drug prices per claim. However this increase is only about 1.2% per year; much less than the pharmacy inflation rates at that time which were about 7.5% per year (7.3% in 2011 to 2012, & 8.0% in 2012 to 2013).[18]

Our results showed a slower PD pharmaceutical price increase than was reported by the Workers’ Compensation Insurance Rating Bureau of California (WCIRB) in 2016 for all claims,[19] and the 17.6% increase in workers’ compensation, drug costs/claim reported from 2010 to 2011.[20] Increases in the cost/claim of PD drugs can be driven by changing patterns of prescriptions that shift treatment to newer, more expensive drugs. Further studies are needed to confirm if the increases in CAWCS drug prices/claim are slowing, as we show.

### Medi-Cal as a primary benchmark

We found that the Medi-Cal fee-schedule has prices for the greatest percentage of PD drugs than any alternative pricing benchmark in the years that we analyzed, which should highlight it as one of the best pricing benchmarks for CAWCS. However, we also found that this percentage decreased over time becoming progressively more limited in the number of drug prices available for Medi-Cal as the primary-fee-schedule. Therefore, Medi-Cal may no longer be an ideal primary fee-schedule for CAWCS.[3,11]

As a result and based on our analysis, we recommend two different approaches for implementing alternative pricing mechanisms. First we recommend that CAWCS could maintain the Medi-Cal fee-schedule as the primary payment mechanism and adopt one of the alternative pricing benchmarks as a secondary payment mechanism when a drug is not in the Medi-Cal fee-schedule. Secondly, we recommend alternative pricing benchmarks to the Medi-Cal fee-schedule as the primary payment mechanism, along with alternative secondary payment mechanisms. First, each pricing benchmarks value as a primary and a secondary fee-schedule benchmark is discussed based on the availability of prices. Next their WC based prices to remain budget neutral are compared.

### AWP as a pricing benchmark

Over three years, 93% of CAWCS drugs had an AWP price in the PD claims, presenting as an ideal primary replacement for the CAWCS fee-schedule pricing benchmark. However, most payers are moving away from AWP pricing because it is subject to artificial inflation and has little relationship to actual wholesale prices. Therefore, we suggest that AWP is a poor choice for CAWCS as a primary price benchmark, despite its having the greatest number of available prices. For CAWCS drugs that had no Medi-Cal prices in the PD claims, AWP provided a price 97% of the time and these percentages increased over time. In 2013, with AWP repricing, CAWCS would have paid AWP-40% (excluding the dispensing fee) for the claims that had a price in the Medi-Cal fee-schedule to remain budget neutral, and AWP– 70% for claims with no Medi-Cal price. These AWP payments were much lower than what CAWCS is currently required by regulation to pay for drugs that have no prices in Medi-Cal, which is AWP-17%.[21] AWP could be a secondary benchmark because it has the highest percentage of drug price coverage for drugs that have no prices under the current fee-schedule and already is the current benchmark that is used for pricing the CAWCS fee-schedule when Medi-Cal does not provide a price. However, if the use of AWP is continued as the secondary benchmark we recommend using at least AWP-40% formula for payment to maintain better budget neutrality.

### NADAC as a pricing benchmark

During the study period, 94% of CAWCS drugs had a NADAC price in the PD CAWCS claims, making it a strong candidate for an alternative primary pricing benchmark for CAWCS. NADAC is focused on outpatient prescriptions and over-the-counter prices from retail community and independent pharmacies,[22] explaining its high percentage of overall PD drug prices available. NADAC provided a price only 24% of the time for CAWCS drugs that had no Medi-Cal prices in the PD claims and should therefore not be used as secondary price benchmark. We recommend that NADAC be used as an alternative to Medi-Cal as the primary benchmark for all PD CAWCS claims; paying NADAC+36% to remain budget neutral, plus a dispensing fee.

### WAC as a pricing benchmark

Only 58.4% of PD CAWCS paid claims had WAC prices in 2011, and although this increased to 80% in 2013, we do not recommend WAC as the primary pricing benchmark as it provides fewer prices than Medi-Cal and NADAC. WAC provided the most prices though (after AWP) for those NDCs with no Medi-Cal price (63%), making WAC one of the best secondary price benchmarks. In 2013, CAWCS would have paid WAC–3% plus a dispensing fee for the 80% of drug NDCs without a Medi-Cal price to remain budget neutral using WAC prices. Pharmaceutical companies typically sell drugs to wholesalers at a list price, WAC or WAC–(1 or 2%).[23] WAC-3% is the discount rate received by wholesalers, and is also our recommendation for a secondary benchmark price. WAC is accessible, administratively simple, has low administrative cost, and is more transparent than AWP. Like AWP, the disadvantage of WAC is that it is not a transaction price and it is therefore also subject to inflation by manufacturers.[11,12] Wisconsin follows the formula of WAC-3.8% for generic and WAC+2% for brand drugs dispensed by retail pharmacies.[17,24,25] We suggest that WAC be used as the second best secondary pricing benchmark, after AWP.

### FUL as a pricing benchmark

Between 2011 and 2013, 74% of CAWCS drugs had FUL prices in the CAWCS PD claims, similar to WAC and so we do not recommend FUL as a primary pricing benchmark. For CAWCS drugs that have no Medi-Cal prices in the PD claims, FUL provided a price for only 15% of claims. This is not surprising since only generic products (at least 3 generics considered) have an FUL price. If brand-name drugs have no generic equivalent an FUL price would not be assigned.[26] This suggests that the majority of drugs with no prices in the current CAWCS fee-schedule are branded drugs. There are three states that use FUL prices as ceiling pricing benchmarks of reimbursement for Medicaid PD drugs; Florida, Tennessee (for specialty pharmacy rates), and Utah.[24] FUL can be used as a ceiling price for multiple-source drugs, but has fewer prices than other fee-schedules so we do not recommend its use for CAWCS for either primary or secondary drug pricing.

### DP as a pricing benchmark

Over three years, 14% of CAWCS drugs had DP prices in the PD CAWCS claims and DP prices were available for only 21% of CAWCS drugs that have no Medi-Cal prices in the PD claims. Therefore, we cannot recommend DP for a primary or secondary pricing benchmark. In 2013, CAWCS paid DP+4% to remain budget neutral for the small number of drugs that could be priced. North Dakota and Maryland follow the formula of DP + 8% as an alternative pricing benchmark for drugs dispensed by retail pharmacies,[24,25] but our analysis found better alternatives.

## Recommendations

The use of the current Medi-Cal fee-schedule as the primary CAWCS fee-schedule for PD drugs is better than all alternatives because it had prices for the largest percentage of drugs compared to all other pricing benchmarks. NADAC also has prices for a high percentage of CAWCS drugs, and because the available Medi-Cal prices may be decreasing, NADAAC may be the best alternative primary fee-schedule for CAWC. Any of the fee-schedule reimbursement approaches however, require combinations of pricing benchmarks. We recommend (Table 4):

1. If Medi-Cal continues to constrict its available fee-schedule prices, we suggest adapting the Delaware method by using 100% of NADAC which covers prices for the next most number of claims after Medi-Cal and AWP. Then for those drugs not covered by the NADAC fee-schedule, we suggest calculating the maximum fee as 60% of AWP of the lowest therapeutically equivalent drug. Finally, for drugs that are not covered by either NADAC or AWP, we suggest calculating the maximum fee as 97% of WAC of the lowest therapeutically equivalent drug. The implications of this choice is more cost-saving than the current CAWCS pricing scheme with estimated cost savings of $17.1 million over the three years.

2. Our second recommendation for CAWCS is to keep the primary reimbursement at 100% of Medi-Cal. For drugs without a Medi-Cal price we suggest continuing with AWP as the secondary pricing benchmark but calculating the maximum fee as 60% of AWP of lowest therapeutically equivalent drug for those payments. To remain budget neutral CAWCS would need to pay 100% Medi-Cal and AWP-75% but since AWP -40% is the best price for brand drugs defined by CBO we suggest using a more reasonable AWP-60% instead. This results in an increase in the budget by $7,103,330, however, so AWP-75% could be considered for pricing the generic drugs to moderate this increase. Finally, for drugs not covered by either Medi-Cal or AWP (which is only 0.09%), we suggest calculating the maximum fee as 97% of WAC of the lowest therapeutically equivalent drug.

3. Our third recommendation for CAWCS pricing is to use the lower of Medi-Cal or NADAC. Then for those drugs not covered by either Medi-Cal or NADAC, we suggest calculating the maximum fee as 60% of AWP of the lowest therapeutically equivalent drug. The implications of this choice would be more cost-saving than the current CAWCS pricing scheme, with estimated cost savings of $17.5 million. Physician dispensed/administered drugs, compounded drugs, and medical foods have been analyzed separately by our research team and have different unique policy issues and require different alternatives. The analysis reported here support our specific recommendations for PD drugs.

**Table 4:** Final recommendation.

*Recommendation 1*: Reimbursement at 100% of Medi-Cal and for drugs are not covered by a Medi-Cal, calculate the maximum fee as 60% of AWP. For drugs that are not covered by either Medi-Cal or AWP, calculate the maximum fee as 97% of WAC				
**100% of Medi-Cal**	97.30%	$109,657,753	$111,627,060	$1,969,307
**60% of AWP**	96.70%	$5,325,626	$10,611,632	$5,286,006
**97% of WAC**	0%	$0	$0	$0
**Drugs that are not covered by the previous pricing benchmarks**	0.1%	$151,983	$0	$151,983
**Total**	99.9%	$115,135,363	$122,238,692	$7,255,313
*Recommendation 2*: Reimbursement at 100% of and for drugs are not covered by NADAC, calculate the maximum fee as 60% of AWP. For drugs that are not covered by either Medi-Cal or AWP, calculate the maximum fee as 97% of WAC				
**100% of NADAC**	94.20%	$101,092,760	$76,869,650	-$24,223,110
**60% of AWP**	88.30%	$12,259,281	$17,625,668	$5,366,387
**97% of WAC**	12.30%	$113,513	$117,532	$4,019
**Drugs that are not covered by the previous pricing benchmarks**	0.6%	$1,669,807	$0	$1,669,807
**Total**	99.4%	$115,135,363	$94,612,850	-$17,182,897
*Recommendation 3*: lower of Medi-Cal or NADAC. Then for those drugs not covered by either Medi-Cal or NADAC, calculate the maximum fee as 60% of AWP				
**lower of Medi-Cal or NADAC** 93.6%		$100,196,486	$75,739,896	-$24,456,486
**60% of AWP** 89.5%		$13,155,473	$18,243,674	$5,088,201
**Drugs that are not covered by the previous pricing benchmarks**	1%	$1,783,408	$0	$1,783,408
**Total**	99%	$115,135,363	$93,983,570	-$17,584,877

Note: CAWCS, California Workers’ Compensation System; AWP, Average Wholesale Price; WAC, Wholesale Acquisition Cost; NADAC, National Average Drug Acquisition Cost.

This table examines the implications of each recommendation and calculates the cost-saving compared to the current CAWCS pricing scheme over three years.

### Limitations

These results are based on CAWCS data between 2011 and 2013, the latest complete data available, but more recent data would be helpful in identifying if Medi-Cal fee-schedule list prices continue to decline as our data suggest. Our data base also included claims that were duplicates, negative payments, and zero payments. Although we worked to eliminate these payments from our analysis, the accuracy of our decisions about these claims provides uncertainty to the estimated total CAWCS drug costs and might change our budget analyses. We feel we handled these factors similarly to other analyses of large claims data bases such as Medicare (personal Communications, September, 2015). Our results, although a good example of pricing alternatives, may not be generalizable to other states’ WC systems or other public or private payers because each has different prescribing and reimbursement practices. We suggest that further study could focus on more detail on the types of drugs that are not benchmarked under the current and alternative fee-schedules to better determine the reasons behind these differences.

## Conclusion

This study focuses on pricing alternatives for systems using a fee-schedule, and how to adopt these alternatives while remaining budget neutral. Our analysis allows us to consider the implications of changing from the current Medi-Cal primary pricing benchmark followed by AWP-17 as the secondary benchmark to an alternative NADAC fee-schedule. It also allows comparison among all pricing alternatives using the CAWCS data to compare their pricing with each other. We suggest that adopting one of the governmental pricing benchmarks such as Medi-Cal and NADAC as the primary pricing benchmarks is the best way to obtain the most complete coverage of the drug claims specific to CAWCS while either remaining budget neutral or lowering total payments. Any governmental pricing benchmark is subject to maintaining price controls by federal agencies. This may be preferable to adopting one of the private pricing benchmarks such as AWP, WAC and DP that could leave CAWCS with a large pharmaceutical bill in the future, if these prices are allowed to rise without oversight. Since all private pricing benchmarks are not representative of actual transactions, are subject to inflation, and difficult to audit, we suggest that it is safer over time to adopt one of the governmental pricing benchmarks as the primary benchmark in a fee-schedule drug system.

CAWCS’s current Medi-Cal fee-schedule has the highest number of prices for PD drugs than all alternatives fee-schedules. However, since Medi-Cal coverage is decreasing over time, we suggest several alternative reimbursement approaches that would price the most CAWCS PD drugs and calculate the total costs of their adoption. All alternatives likely require combinations of pricing benchmarks but can provide continued access and even savings to CAWCS. A fee-schedule has limitations for providing a full range of access and price control techniques. Another alternative that is currently being considered by CAWCS, that is not directly addressed in our study, is adopting a formulary approach for drug payment rather than using a fee-schedule. The adoption of an evidence-based formulary has been requested for CAWCS by the end of 2017 and rule-making is in progress at the time of this writing with expectation of a January 1, 2018 start date.[27]

CAWCS currently has a very permissive coverage of pharmaceuticals, with almost no restrictions on drug coverage. This type of mechanism makes controlling drug prices difficult, and dependent on demand-based approaches. The adoption of a drug formulary would open up the CAWCS to the types of incentive based supply-side approaches to controlling pharmaceutical use and costs and may even improve the quality of drug use. Supply-side approaches include offering suggestions for preferred drugs based on quality targeted to specific patient needs, as well as costs. In an era of personalized medicine, CAWCS should consider the formulary approach, which might align drug use better with their current Medi-Cal pricing benchmark. Alternatively, they can keep using a fee-schedule and adopt one of the alternative pricing benchmarks suggested by our study: using NADAC as their primary pricing benchmark or using AWP-40% or DP as secondary pricing benchmarks while retaining Medi-Cal as their primary benchmark.

## Supporting information

S1 Dataset (XLSX)
